# Oxidative Stress and Inflammation Induced by Waterpipe Tobacco Smoking Despite Possible Protective Effects of Exercise Training: A Review of the Literature

**DOI:** 10.3390/antiox9090777

**Published:** 2020-08-21

**Authors:** Behzad Taati, Hamid Arazi, Katsuhiko Suzuki

**Affiliations:** 1 Department of Exercise Physiology, Faculty of Sport Sciences, University of Guilan, Rasht 4199843653, Iran; taati.behzad@yahoo.com; 2 Faculty of Sport Sciences, Waseda University, Tokorozawa 359-1192, Japan; katsu.suzu@waseda.jp

**Keywords:** hookah, shisha, ghalyan, cigarette smoking, chronic diseases, reactive oxygen species, aerobic training, exercise capacity, lung function

## Abstract

The prevalence of waterpipe tobacco smoking (WTS), which is also known as ghalyan, shisha or hookah, is increasing rapidly around the world, especially among youth. Growing interest in this form of tobacco smoking can be traced, in part, to the use of flavored tobacco products, social acceptability as a safer option than cigarettes, and its consideration as a relaxation method or entertainment. However, there is a well-established association between WTS and oxidative stress that causes irreversible chronic pathological conditions such as cardiovascular and respiratory problems, as well as different types of cancers, and thus increases the risk of mortality. Clearly, induction of inflammation status through increased reactive oxygen species (ROS), which in turn leads to oxidative stress and harm to lipids, DNA, and proteins, is the most plausible mechanism to explain the potential harmful effects of WTS. Unlike WTS, well-designed exercise training programs increase ROS to the extent that it is beneficial to the body. In this study, we aimed to review available evidence on the impact of exercise training on oxidative stress and inflammation status. We also summarize the effect of acute and chronic WTS on different exercise capacities.

## 1. Introduction

The consumption of tobacco is one of the most serious public health problems the world has ever faced, with more than 8 million deaths a year that can be directly attribute to tobacco smoking or are a result of being exposed to second-hand smoke [1,2]. It is also predicted that at least 1 billion will die the 21st century if current trends in use persist [3]. Tobacco products are defined by the World Health Organization (WHO) as “products entirely or partly made of the leaf tobacco as raw material which are manufactured to be used for smoking, sucking, chewing or snuffing” with about 1.3 billion users worldwide according to the earlier estimates [2]. Of these, cigarettes are the most favored product (82%), as more than six trillion cigarettes are produced annually and about one billion smokers consume these products in the world [1,3]. However, the increasing trend of other forms of tobacco use is yet to be addressed.

Waterpipe tobacco smoking (WTS), which is popularly referred to as “ghalyan”, “narghile”, “shisha”, “hookah”, or “hubble-bubble”, involves the use of a multi-stemmed device that allows tobacco smoke passes through a bowl of water prior to inhalation [4,5]. Growing evidence suggests that prevalence of WTS is increasing in many parts of the world, especially in the Eastern Mediterranean (7.25%), the Middle Eastern (6% to 34%) and the Americas (3.8%), with an alarming prevalence among the youth (school and university students) and women [5,6,7,8,9]. Even though the people who use waterpipe are aware of the health risks of WTS, they perceived it as less hazardous and less addictive than cigarette smoking [10]. Indeed, at least part of the popularity of WTS is due to the common misperception that the water will filter the smoke and thus this method of tobacco smoking is safer than cigarettes [11,12]. On the other hand, socializing, relaxation, pleasure and entertainment are considered as the main motives for WTS popularity [10].

Currently, it is well-documented that WTS on both a short- and long-term basis is probably associated with a variety of adverse health outcomes including cardiovascular and respiratory disease, several types of cancer and an increased risk of mortality [13,14,15]. Moreover, there is overwhelming evidence that the impact of WTS on inflammatory responses and antioxidant status is similar or even worse than that of cigarette smoking [11,12,16,17,18,19]. In this context, Khabour et al. showed that acute (7 days) [11] and chronic (6 weeks) [16] exposure to waterpipe tobacco smoke induced significant alterations in inflammatory cytokines, oxidative stress markers and absolute count of macrophages, lymphocytes and neutrophils in the lung of mice. In general, it can be stated according to the relevant literature that WTS-induced oxidative stress that initiates local and systemic inflammation status is the main mechanism of its deleterious health effects [11,12,16,17,18,19,20].

While skeletal muscle contractions—for example, during exercise training—are a generating source of reactive oxygen species (ROS), which are necessary for normal physiological functions according to hormesis phenomenon [12,21], WTS produces large amounts of ROS that have adverse reactions to lipids, DNA, and proteins [22]. It is generally agreed that regular exercise training with the appropriate intensity and duration improves immune functions, such as recirculation of neutrophils and anti-inflammatory cytokines and increasing the functional activity of tissue macrophages, through anti-inflammatory and antioxidant effects [23,24,25,26]. Moreover, single bouts of moderate-intensity exercise can also induce these effects that add up over time to strengthen immune defense [23,26]. Therefore, the present review aimed to summarize the current knowledge about: (1) the effect of short- and long-term exercise training on oxidative stress and inflammatory biomarkers induced by WTS; (2) impact of WTS on exercise capacity in waterpipe users.

## 2. An Overview of Waterpipes

**Figure 1:**
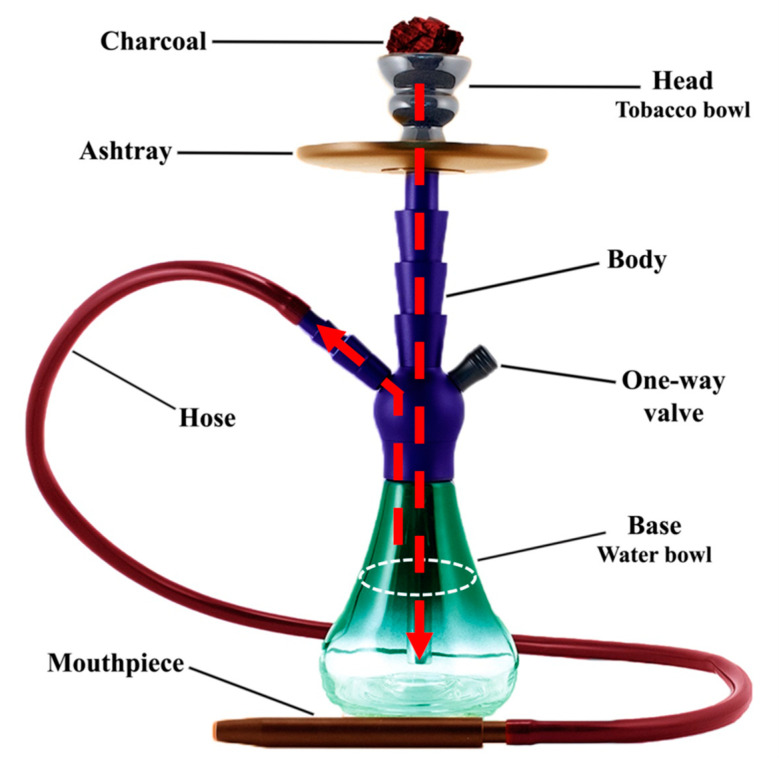
Different parts of one of the most common types of waterpipe smoking device.

Regional differences in some waterpipe design characteristics (such as head or water bowl size, number of mouthpieces, the height of the body, etc.) can be observed, but all of them contain a kind of liquid (such as water, milk, alcohol, etc.) through which smoke passes prior to reaching the smoker. Waterpipe devices typically consist of a head, a wooden or metal body, a base, and a slender, flexible hose (Figure 1). A glass, marble, or clay bowl that is half full of water is placed at the base. The head is a clay, metal, or ceramic bowl containing tobacco, which is separated from the charcoal or a briquette by a perforated aluminum foil sheet. When the smoker inhales through the hose, a vacuum creates above the water and draws air throughout the body of waterpipe, tobacco bowl and lit coal. Therefore, indirect heat hits the tobacco and a mixture of the coal and tobacco smoke passes from the holes located in the bottom of the head into the body’s central conduit, which is submerged in water. Produced smoke then reaches the smoker by the plastic or leather hose that terminates with a mouthpiece. The hose is not submerged and connects to the top of the water bowl [4,14].

## 3. Toxicants Released from Waterpipe Smoking

The use of flavored and sweetened tobacco, known as “Maassel”, was introduced in the 1990s. Today, these tobacco types, especially tobacco with a fruity flavor including double apple, orange, peach, cherry, grape, etc., are believed to have made WTS more popular among adults and youth in both sexes [5]. The interesting point is that although some stems and glycerin use to aid in fermentation and produce less nicotine-rich tobacco, Maassel does not have less nicotine content compared to cigarettes [13,27]. In fact, the consumption of Maassel using WTS has various types of potentially harmful substances that are quite similar to those in cigarette tobacco [28]. However, there are some differences in the relative amounts of these toxicants, combustion mode, burning temperature and smoke volume delivered. Moreover, the consumption of one cigarette lasts about 5 to 7 min with 8 to 12 puffs (40 to 75 mL) and the smoker typically inhales 0.5 to 0.6 L of smoke. In contrast, each typical run or session of WTS needs more time (20 to 80 min), with about 50 to 200 puffs which range from about 0.15 to 1 L each [13,15,28,29]. Therefore, the smoke volume inhaled from a single session of WTS may be similar to consuming 100 or more cigarettes [15].

There are numerous compounds in tobacco smoke in which nicotine, carbon monoxide (CO), arsenic, volatile organic chemicals (VOCs), particulate matter (PM), heavy metals, acrolein, and various carcinogens are identified to have served as the main toxicants [13,14]. Generally, the waterpipe smoker use much more amounts of tobacco per one session of WTS versus one cigarette. However, it should be noted that this is a routine procedure in this method of tobacco smoking and lesser amounts of tobacco are not consumed during a standard session of WTS.

Table 1 shows the differences of the most well-known pollutants in smoking one cigarette relative to one session of WTS. In comparison to smoking one cigarette, WTS is associated with greater CO, dramatically more smoke exposure, and similar nicotine content [27]. Nevertheless, it is estimated in another study that one session of WTS significantly increases the plasma levels of nicotine in a way that is equivalent to smoking 2–3 cigarettes [30], and this may also increase to more than 9 mg per session if the waterpipe device is used without water [31]. Many toxicants from WTS are found to originate from charcoal. For example, Benzo[a]pyrene is a polycyclic aromatic hydrocarbon and known as a potent carcinogen that decreases to a considerable amount by replacing the burning charcoal with an electric heater [32]. It has been proposed that the charcoal used for WTS is the main source of heavy metals including lead (Pb) and chromium (Cr) [33]. The use of lit charcoal in WTS can also lead to generate three to ten times more CO to the smokers than smoking a single cigarette so that produced CO was reduced by 90% when charcoal was substituted with electrical heating to heat up the tobacco [13,32]. In addition, the material used for the hose (leather or plastic) is another important factor in CO emission by WTS [34]. Interestingly, the exhaled CO after even an entire pack of cigarettes is lower than the amount exhaled after a WTS session [35].

## 4. Waterpipe Smoking, Oxidative Stress and Inflammation

The underlying mechanisms responsible for adverse health effects of WTS might be attributed to induction of oxidative stress and inflammation. Oxidative stress is a situation in which there is a transient or chronic imbalance between the amount of ROS and the antioxidant capacity, affecting the homeostasis of the redox state and leading to local and systemic inflammation [12,13].

**Figure 2:**
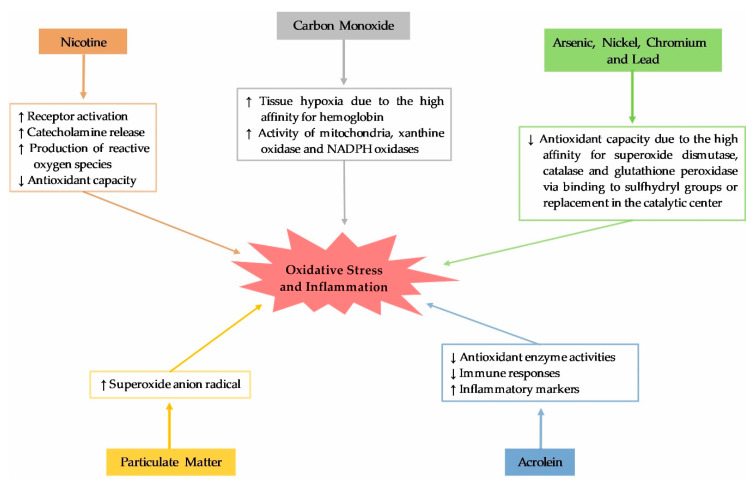
The most important toxicants in waterpipe tobacco smoking that induce oxidative stress and inflammation.

Tobacco smoking is one of the most common environmental factors related to the accumulation of ROS that can be deleterious. As shown in Table 1, there are many similarities between the profile of common toxic substances found in WTS and cigarette smoking. Therefore, it has been expressed that many of the health consequences of these two tobacco products can also be similar [20,27]. Figure 2 shows some of the most threatening constituents found in waterpipe smoke and their action mechanisms related to oxidative stress and inflammation. The amount of nicotine and CO inhaled in one session of WTS is remarkable. Receptor activation and catecholamine release, and also induction of oxidative stress either by increasing ROS production or decreasing antioxidant capacity are the major pathways for the pathological effects of nicotine [13,48,49]. For instance, nicotine exposure increased renal oxidative stress parameters and expression of the pro-oxidant SHC-transforming protein (p66Shc), and also suppressed expression of the antioxidant superoxide dismutase (SOD) in mice [49]. In contrast, because the affinity of CO for hemoglobin is 250 times greater than for oxygen, CO inhalation can lead to tissue hypoxia as a result of readily absorption in the lungs and formation of a tight but reversible bond with hemoglobin [50]. Tissue oxygen reperfusion after CO elimination results in increased activity of mitochondria, xanthine oxidase and NADPH oxidases, which produces a burst of ROS from these sources [51].

Heavy metals, VOCs, and PM present in waterpipe smoke are other potential factors involved in aggravating oxidative stress and inflammation. Heavy metals such as arsenic, nickel, Cr and Pb may negatively affect antioxidant defense system [13]. As a result of binding to sulfhydryl (SH) groups, the metals have high affinity for antioxidant enzymes such as SOD, catalase and glutathione peroxidase (GPx), and also small molecular antioxidants (e.g., glutathione) [13,52]. Moreover, heavy metals can interact with these enzymes through replacement of metals ions in the catalytic center [52]. In addition to the metals, WTS is a dominant source of VOCs. For example, acrolein, as a potent aldehyde, increases ROS production and lipids peroxidation via decreasing antioxidant enzyme activities, suppresses immune responses and enhances inflammatory markers [53]. On the other hand, PM is widely believed to affect redox regulated pathways [54]. In this case, significant increases in superoxide anion radical in rats exposed to PM_2.5_ (as one of the most important size-fractions of PM) for 10 weeks are detected [55].

In general, clinical and experimental research on WTS effects provides evidence of increases in oxidative stress and inflammation. Similar to cigarettes, short-term exposure to waterpipe smoke (1 h/day for 7 days) is found to be harmful to the lungs and airways due to the elevations in total white blood cell count, absolute count of neutrophils, monocytes and lymphocytes, pro-inflammatory cytokines, and oxidative stress markers [11]. Likewise, lipid peroxidation levels in lung tissue, total inflammatory cells, tumor necrosis factor α (TNF-α), interleukin (IL)-6, IL-1β and IL-13 increased, whereas those of IL-10 were reduced by 4 to 6 weeks of exposure to waterpipe smoke in animal models [16,17,19]. Moreover, chronic WTS exposure significantly decreased anti-oxidative markers in the lung tissue such as SOD and glutathione (GSH) [19], but these findings were not confirmed by other studies [16,17].

WTS also has destructive effects on the kidney and heart parameters. One month of exposure to waterpipe smoke has shown significant increases in kidney thiobarbituric acid reactive substances (TBARS), blood urea nitrogen (BUN) [56], serum creatinine, oxidized GSH (GSSG) [56,57], kidney ROS generation and lipid peroxidation, proteinuria, urinary kidney injury molecule-1 (KIM-1), renal concentrations of IL-6, IL-1β and KIM-1 [57], and also significant reductions in catalase [56,57], SOD and GPx kidney activity in mice [56]. Likewise, short-term nose-only exposure to mainstream WTS (30 min/day for 5 consecutive days) exerts cardiac inflammation and oxidative stress [18].

## 5. Impact of Exercise Training on Oxidative Stress and Inflammatory Markers in Waterpipe Smokers

### 5.1. Acute Responses after Exercise

Long-term WTS has been shown to be associated with an impaired antioxidative response and greater hematological indices potentially reflective of greater inflammation following acute exercise [12,58]. In this context, we previously observed that the responses of salivary antioxidative markers including peroxidase (POX) activity (about 9%) and the percentage of scavenging activity against 2,2-diphenyl-1-picryl-hydrazyl-hydrate (DPPH) radical (about 7%) after a single bout of exhaustive aerobic exercise (Bruce protocol treadmill test) were weaker in waterpipe smokers than those of non-smokers [12]. It has also been reported that a 30-s Wingate supramaximal exercise test resulted in significant greater increases in immune cell counts including white blood cells, neutrophils, and lymphocytes in waterpipe smokers relative to non-smokers [58]. However, future studies need to elucidate the impact of long-term WTS on human antioxidants, oxidative stress and inflammatory responses after acute exercise training.

### 5.2. Effects of Regular Exercise Training

On the basis of pooled data from animal [8,59,60] and human [61,62] studies (Table 2), regular exercise training significantly alleviates pro-inflammatory and oxidative effects elicited by WTS. Duration of training protocols in these trials ranged from 4 to 12 weeks and the type of exercise performed was aerobic training including swimming and running. Findings from animal studies show that 1 h/day moderate-intensity swimming exercise 5 days/week for 4 weeks improved the activity of catalase, GPx, and glutathione/oxidized glutathione ratio in the hippocampus [59], and also reduced TNF-α levels, and normalized the activity of catalase enzyme in the heart [8]. It should be noted that all these parameters had been impaired by WTS exposure. Despite the swimming protocol not normalizing the levels of other pro-inflammatory (IL-1β and IL-6) and anti-inflammatory (IL-10) cytokines induced by WTS in the heart [8], a moderate-intensity running protocol (40 min/day, 5 days/week) for a longer duration (8 weeks) significantly abrogated the latter augmentation of IL-6 in lung homogenate [60]. Likewise, the concentrations of TNF-α, 8-isoprostane, and intra-alveolar macrophages, together with airway resistance, lung DNA damage, and focal damage to alveolar septae caused by WTS were significantly decreased in trained mice [60]. These results suggest that WTS induces anti-oxidative scavenging dysfunction and inflammation status in the heart, brain, and lungs, while at least 4 to 8 weeks of mild aerobic training are needed to achieve significant improvements in such alterations.

Human studies have also confirmed that aerobic training may be very beneficial in the defense and prevention of WTS-dependent oxidative stress and inflammation. In detail, the reports of two research articles published by the same research team in 2015 indicate that both modality of a 12-week aerobic training program including moderate-intensity interval training (MIIT) and low-intensity continuous training (LICT) have an important role in oxidative stress attenuation in waterpipe male smokers [61,62]. The LICT protocol was 20 to 30 min of running, three times per week at an intensity of 40% of maximum oxygen uptake (VO_2max_), and the MIIT consisted of 2-min intervals of running interspersed with recovery periods of 1 min, three sessions per week lasting 30 min at an intensity of 70% of VO_2max_. Both exercise protocol appeared to increase plasma total antioxidant status (TAS), SOD, GPx, α-tocopherol, glutathione reductase (GR) and decrease malondialdehyde (MDA) in waterpipe smokers [61,62].

To date, there is no certain mechanism to explain how regular exercise training is able to exert its modulatory effects on outcomes of WTS. Nonetheless, it has been advocated that waterpipe smoke exposure leads to a significant increase in the expression of nuclear factor kappa-B (NF-κB), and the protective effect exerted by exercise training is, at least partly, related to the suppression of NF-κB expression and the facilitation of activating nuclear factor erythroid 2-related factor 2 (Nrf2) signaling pathways [60]. While Nrf2 has long been considered a central element of antioxidant regulation in cellular systems, strong evidence suggests that NF-κB pathway has a key role in the expression of pro-inflammatory genes including cytokines, chemokines, and adhesion molecules [63,64]. When ROS production increases during exercise training, endogenous and exogenous antioxidant defenses may be unable to control these changes. Therefore, consequent oxidative stress triggers the activation of the transcriptional factor Nrf2 to provide the antioxidant response [63]. It has been established that impaired Nrf2 expression reduces exercise performance, energy expenditure, mitochondrial volume and antioxidant activity following exercise training [65]. Importantly, both acute and chronic exercise training have been consistently found to activate Nrf2 signaling across multiple tissues and species [66]. Regular exercise training can also inhibit IκBα/NF-κB signaling pathway which results in the down-regulation of inflammatory genes such as IL-6 and TNF-α [67].

The expression of peroxisome proliferator-activated receptor gamma coactivator 1-alpha (PGC-1α) during exercise training is another transcription factor that is probably responsible for antioxidative effects of exercise training as a result of enhancing antioxidant defenses against exacerbated ROS generation [68]. In fact, each bout of exercise leads to an accumulation of PGC-1α through which these beneficial effects could be achieved. Therefore, current data suggest PGC-1α as a central player in orchestrating many of the oxidative adaptations to exercise training [69]. Generally, understanding the precise mechanisms by which exercise training programs confer protection against WTS harms requires further well-designed studies with an adequate sample size.

## 6. Waterpipe Effects on Exercise Capacity and Lung Functions

WTS can negatively affect exercise capacity (Table 3). In fact, even acute WTS (a 45-min session) could decrease oxygen pulse and VO_2_ during a cardiopulmonary exercise test and also increase rating of perceived exertion (RPE, measured by Borg scale) at mid and peak level of the exercise [70]. Concerning long-term WTS, it has been demonstrated that VO_2max_, as a common and valid measure of aerobic capacity, is lower in waterpipe smokers than non-smokers [61,62,71,72]. Moreover, it seems that waterpipe smokers reach exhaustion earlier in a given incremental exercise test [73], and heart rate recovery after exercise testing needs more time in waterpipe smokers relative to non-smokers and cigarette smokers [72].

On the other hand, as shown in Table 3, WTS is associated with a decline in pulmonary functions so that different respiratory parameters in waterpipe smokers, such as forced vital capacity (FVC), forced expiratory volume in one second (FEV_1_), peak expiratory flow (PEF), total lung capacity (TLC), and forced expiratory flow at 50% of FVC (FEF_50%_), were significantly lower than those of non-smokers [71,72,73,74]. In addition to long-term effects, a single session of WTS appears to induce impairment in lung function (e.g., the amount of FEF_25–75%_) [70].

Regardless of these undesirable changes, regular exercise training may enhance cardiorespiratory fitness and mitigates lung function decline caused by WTS [72]. In two different research articles in this regard, Koubaa et al. investigated the effect of two modality of a 12-week running training programs, 3 days/week, including MIIT (30 min of interval exercise, 2 min of work followed by 1 min of rest, 70% of VO_2max_) and LICT (20–30 min, 40% of VO_2max_) in sedentary male smokers. While LICT protocol improved VO_2max_ values and some respiratory parameters (i.e., FVC, FEV_1_, and FEF_50%_), only percentage of PEF increased by MIIT program [72,74]. Although such improvements were reported as a result of LICT method, there is very little information on the effects of exercise training on various health factors related to short- and long-term WTS.

## 7. Conclusions

It can be concluded, based on the reviewed literature, that WTS is not safer than cigarette smoking in relation to ROS generation and inflammation induction. WTS not only causes an increased generation of free radicals, but also exposes smokers to high levels of potential toxic substances. It seems that WTS users have lower antioxidant capacity in response to exercise and also acute and chronic WTS can reduce exercise capacity. Although we would generally recommend smoking cessation rather than exercise training, people who are unable to quit WTS could follow a well-designed aerobic training protocol (e.g., low-intensity continuous or moderate-intensity interval training) in order to boost their antioxidant defense system and minimize inflammatory responses caused by smoking. Perhaps exercise training reduces adverse changes of WTS, but the balance is certainly shifted towards processes that are unfavorable and dangerous to the health of smokers.

Finally, it is important to draw attention on the need for further studies on the effect of other types of exercise training such as resistance or high-intensity interval training. Furthermore, optimal training variables (i.e., duration, intensity, and frequency) to achieve the best outcomes in WTS users is needed to be examined.

## Figures and Tables

**Table 1:** Absolute and relative amounts of different harmful constituents released from one session of waterpipe tobacco smoking (WTS) versus smoking a single cigarette.

Toxicant	WTS	Cigarette	Approximate Fold Difference (X)
Volatile Aldehydes, µg [36,37,38,39]			
Formaldehyde	58.7 to 630	20.6 to 100	3 to 6
Acetaldehyde	383 to 2520	587.4	0.7 to 4
Propionaldehyde	51.7 to 403	49	1 to 8
Acrolein	892	60 to 240	4 to 15
Acetone	118	270.4	0.5
Volatile Organic Compounds, µg [40,41]			
Toluene	9.92	64.9	0.15
Benzene	271	43.4	6
Isoprene	4	298	<0.1
Heavy Metals, ng [42,43]			
Lead	6870	34 to 85	81 to 202
Chromium	1340	4 to 70	19 to 335
Nickel	990	600	1.5
Arsenic	165	40 to 120	1.5 to 4
Cobalt	70	0.13 to 0.2	350 to 538
Beryllium	65	300	0.2
Carcinogenic polycyclic aromatic hydrocarbon, ng [36,44]			
Chrysene	106	16.2	6.5
Benz(a)anthracene	86.4	14.1	6
Benzo(b + k)fluoranthenes	64.7	7.6	8.5
Benzo(a)pyrene	51.8	6.6	8
Indeno(1,2,3-cd)pyrene	47.3	3.8	12.5
Others [36,37,45,46,47]			
Nicotine, mg	1.04 to 4.82	0.73 to 2.39	1.5 to 2
Carbon monoxide, mg	150 to 155	12 to 22.5	7 to 12.5
Tar, mg	464 to 640	9.4 to 29	22 to 49
Particulate matter, mg	770 to 1193	11	70 to 108
Nitric oxide, µg	437	218.1	2

**Table 2:** Research on the effects of short- and long-term exercise training on inflammatory and oxidative stress outcomes caused by waterpipe tobacco smoking (WTS).

The Authors	Subjects	Purpose	Exercise Protocol	Key Findings
Arazi et al. [12]	Sedentary women (11 waterpipe smokers, 12 non-smokers)	Comparing the salivary antioxidative responses following a bout of exhaustive aerobic exercise	Start at 1.7 mph and a gradient of 10% for the first 3 min, the gradient increased by 2% every 3 min, and the speed was 2.5, 3.4, 4.2, 5, 5.5, and 6 mph in the subsequent stages (Bruce treadmill test)	Smaller increase in POX activity, larger decline in DPPH activity, and lower salivary flow rate for smokers ↔ UA
Ahmadian et al. [58]	Sedentary men (10 waterpipe smokers, 10 non-smokers)	The influence of WTS on cognitive function and hematological parameters following an acute supramaximal exercise	30 s Wingate supramaximal exercise test using a cycle ergometer	Greater increases in white blood cell, neutrophil, hematocrit and lymphocyte values for smokers ↔ PLT, PDW, MPV
Nakhaee et al. [8]	Wistar male rats	The effects of waterpipe exposure with/without swimming exercise on heart histology and inflammation status	5 days/week for 4 weeks, 1 h/day, moderate intensity	↔ MDA, GPX, SOD, IL-10, IL-1β, and IL-6 ↓ TNF-α ↑ Catalase
Alzoubi et al. [59]	Wistar male rats	The neuroprotective effects of swimming exercise on hippocampus oxidative markers induced by exposure to waterpipe	5 days/week for 4 weeks, 1 h/day, moderate intensity	↑ GPX, Catalase, and GSH/GSSG ↓ GSSG ↔ TBARs, GSH
Nemmar et al. [60]	C57BL/6 mice	The impact of regular exercise training on lung inflammation and impairment of pulmonary function induced by exposure to waterpipe	Treadmill running, 5 days/week for 8 weeks, 40 min/day, moderate intensity	↓ TNF-α, IL-6, NF-κB, 8-isoprostane, intra-alveolar macrophages, airway resistance, lung DNA damage, and focal damage to alveolar septae ↑ Nrf2
Koubaa et al. [61]	Sedentary men (12 waterpipe smokers, 11 cigarette smokers, and 12 non-smokers)	The impact of interval training program on the antioxidant defense capability and lipid profile	Race track running, 3 days/week for 12 weeks, 30 min/day, 2-min intervals interspersed with recovery periods of 1 min, moderate-intensity (70% of VO_2max_)	↑ TAS, SOD, GPx, GR, α-tocopherol, and HDL-C ↓ MDA, and TC/HDL-C ↔ LDL-C, TC, TG, HDL-C/TG
Koubaa et al. [62]	Sedentary men (14 waterpipe smokers, 15 cigarette smokers, and 14 non-smokers)	The effect of continuous training program on antioxidant defense capability and lipid profile	Race track running, 3 days/week for 12 weeks, 20–30 min/day, low-intensity (40% of VO_2max_)	↑ TAS, SOD, GPx, GR, α-tocopherol, and HDL-C ↓ MDA, LDL-C, TC, and TC/HDL-C ↔ MDA, GR, α-tocopherol, TG, HDL-C/TG

↑ increase; ↓ decrease; ↔ no change; WTS: waterpipe tobacco smoking; POX: peroxidase; DPPH: 2,2-diphenyl-1-picryl-hydrazyl-hydrate; UA: uric acid; TAS: total antioxidant status; SOD: superoxide dismutase; GPx: glutathione peroxidase; GR: glutathione reductase; GSH/GSSG: glutathione/oxidized glutathione ratio; TBARs: thiobarbituric acid reactive substance; TNF-α: tumor necrosis factor α; MDA: malondialdehyde; IL-6, 10, 1β: interleukin 6, 10, 1β; NF-κB: nuclear factor kappa-B; Nrf2: nuclear factor erythroid 2-related factor 2; PLT: platelets total; PDW: platelet distribution width; MPV: mean platelet volume; HDL-C: high-density lipoprotein cholesterol; LDL-C: low-density lipoprotein cholesterol; TC: total cholesterol.

**Table 3:** Research on the effects of acute and chronic waterpipe tobacco smoking (WTS) on exercise capacity and lung function.

The Authors	Subjects	Purpose	Exercise Protocol	Key Findings
Hawari et al. [70]	24 healthy men	The acute effects of WTS on exercise capacity and lung function	Cardiopulmonary exercise test using a cycle ergometer: 2-min 20-Watt warm up and 25-Watt increase every 2-min for a maximum time of 10 min	↓ VO_2_, O_2_ pulse, FEF_25–75%_ ↑ HR/VO_2_, baseline respiratory rate, RPE at mid and peak exercise ↔ FEV_1_, FVC, DLco, breathing reserve
Koubaa et al. [71]	68 sedentary men (22 waterpipe smokers, 23 cigarette smokers, 23 non-smokers)	Evaluate and compare the effect of smoking on antioxidant status, aerobic capacity, pulmonary function and lipid profile in waterpipe and cigarette smokers	Cardiopulmonary exercise test using a cycle ergometer: 5-min warm up with 6 km/h, 1 km/h increase every 2 min	↓ VO_2max_, MAS, FVC, FEV_1_, PEF, FEF_25–75%_, FEF_50%_ ↔ FEV_1_/FVC
Koubaa et al. [72]	43 sedentary men (14 waterpipe smokers, 15 cigarette smokers, 14 non-smokers)	The effects of continuous training on lungs function and cardiorespiratory fitness in smokers	Race track running, 3 days/week for 12 weeks, 20–30 min/day, low-intensity (40% of VO_2max_)	↑ FVC, FEV_1_, FEF_50%_, VO_2max_, vVO_2max_ ↔ PEF, FEV_1_/FVC, FEF_25–75%_
Koubaa et al. [74]	35 sedentary men (10 waterpipe smokers, 12 cigarette smokers, 11 non-smokers)	The effects of aerobic interval training program on aerobic capacity and pulmonary function in smokers	Race track running, 3 days/week for 12 weeks, 30 min/day, 2-min intervals interspersed with recovery periods of 1 min, moderate-intensity (70% of VO_2max_)	↑ VO_2max_, vVO_2max_, PEF

↑ increase; ↓ decrease; ↔ no change; WTS: waterpipe tobacco smoking; VO_2_: oxygen uptake; VO2max: maximum VO_2_; vVO_2max_: velocity at VO2max; MAS: maximal aerobic speed; RPE: rating of perceived exertion; DLco: diffusing lung capacity; FVC: forced vital capacity; FEV_1_: forced expiratory volume in one second; PEF: peak expiratory flow; FEF: forced expiratory flow; FEF_50%_: FEF at 50% of FVC; FEF_25–75%_: FEF over the middle half of the FVC.
